# A Correlate of HIV-1 Control Consisting of Both Innate and Adaptive Immune Parameters Best Predicts Viral Load by Multivariable Analysis in HIV-1 Infected Viremic Controllers and Chronically-Infected Non-Controllers

**DOI:** 10.1371/journal.pone.0103209

**Published:** 2014-07-31

**Authors:** Costin Tomescu, Qin Liu, Brian N. Ross, Xiangfan Yin, Kenneth Lynn, Karam C. Mounzer, Jay R. Kostman, Luis J. Montaner

**Affiliations:** 1 The Wistar Institute, HIV Immunopathogenesis Laboratory, Philadelphia, Pennsylvania, United States of America; 2 The Wistar Institute, Biostatistics Laboratory, Philadelphia, Pennsylvania, United States of America; 3 UPENN-Presbyterian Medical Center, Philadelphia, Pennsylvania, United States of America; 4 Philadelphia FIGHT, The Jonathan Lax Treatment Center, Philadelphia, Pennsylvania, United States of America; University of Pittsburgh Center for Vaccine Research, United States of America

## Abstract

HIV-1 infected viremic controllers maintain durable viral suppression below 2000 copies viral RNA/ml without anti-retroviral therapy (ART), and the immunological factor(s) associated with host control in presence of low but detectable viral replication are of considerable interest. Here, we utilized a multivariable analysis to identify which innate and adaptive immune parameters best correlated with viral control utilizing a cohort of viremic controllers (median 704 viral RNA/ml) and non-controllers (median 21,932 viral RNA/ml) that were matched for similar CD4^+^ T cell counts in the absence of ART. We observed that HIV-1 Gag-specific CD8^+^ T cell responses were preferentially targeted over Pol-specific responses in viremic controllers (*p = 0.0137*), while Pol-specific responses were positively associated with viral load (rho = 0.7753, *p = 0.0001*, n = 23). Viremic controllers exhibited significantly higher NK and plasmacytoid dendritic cells (pDC) frequency as well as retained expression of the NK CD16 receptor and strong target cell-induced NK cell IFN-gamma production compared to non-controllers (*p<0.05*). Despite differences in innate and adaptive immune function however, both viremic controllers (*p<0.05*) and non-controller subjects (*p<0.001*) exhibited significantly increased CD8^+^ T cell activation and spontaneous NK cell degranulation compared to uninfected donors. Overall, we identified that a combination of innate (pDC frequency) and adaptive (Pol-specific CD8^+^ T cell responses) immune parameters best predicted viral load (R^2^ = 0.5864, *p = 0.0021, n = 17*) by a multivariable analysis. Together, this data indicates that preferential Gag-specific over Pol-specific CD8^+^ T cell responses along with a retention of functional innate subsets best predict host control over viral replication in HIV-1 infected viremic controllers compared to chronically-infected non-controllers.

## Introduction

HIV-1 infected controllers maintain durable viral suppression without anti-retroviral therapy (ART) and have generally been defined as either having undetectable HIV-1 RNA levels using conventional assays (elite controllers) or having low but detectable levels of viral replication below 2000 copies viral RNA/ml (viremic controllers) [1], [2], [3], [4], [5], [6]. Although mechanisms of elite control have been widely studied [7], [8], [9], [10], [11], [12], [13], the immunological factor(s) associated with host control in presence of low but detectable viral replication in viremic controllers remains of considerable interest.

Inheritance of protective MHC Class I (MHC-I) alleles, important for CD8^+^ T cell recognition of target cells (such as HLA-B*57), has been associated with delayed progression to AIDS [14], [15], [16], [17] and found to be enriched in HIV-1 controller cohorts [18], [19]. CD8^+^ T cell responses directed against the HIV-1 Gag protein are increased in individuals with protective MHC-I alleles [20], [21], [22], [23], [24], [25] and are associated with lower viral loads [26], [27], [28]. Gag-specific CD8^+^ T cell responses target virally infected target cells early in the viral life cycle before integration and viral replication occurs [29], and are believed to limit viral replication by targeting conserved epitopes that reduce viral fitness following the emergence of escape mutations [30], [31], [32], [33]. Although a role for the adaptive T cell response in maintaining low viral loads among controller subjects is supported by these studies, as many as half of the subjects from HIV-1 controller cohorts exhibit low to undetectable Gag-specific CD8^+^ T cell responses and/or lack protective MHC-I alleles [18], [19].

There is an increasing body of literature that supports the hypothesis that innate immune responses may also contribute to sustained viral control without ART during HIV-1 infection. Genetic data has shown a consistent association between certain NK cell Killer Inhibitory Receptor alleles of the KIR3DL1 locus with lower viral loads and/or delayed progression to AIDS [34], [35], [36]. In vitro, NK cells expressing these protective NK alleles have been shown to produce more IFN-gamma [37], have increased poly-functionality [38], [39], and mediate stronger inhibition of HIV-1 replication [40]. The function of both NK cells and plasmacytoid dentritic cells (pDC) is retained in long-term non-progressors and elite controllers [10], [39], [41]. Recently, we have shown that increased NK activity can be evidenced in the absence of strong HIV-1 Gag-specific CD8^+^ T cell responses in HIV-1 infected elite controllers [42]. Together, these data suggest that the innate immune response may account for an additional level of immune control over HIV-1, yet no study has modeled viral load utilizing a combination of both innate and adaptive immune parameters.

The identification of the immunological mechanisms that segregate viremic controllers with viral loads below 2,000 copies viral RNA/mL from non-controllers with high viral loads are typically hampered by gross disparities in CD4^+^ T cell count and anti-retroviral therapy status between the two groups. Here, we assessed the phenotype, activation status and function of peripheral blood NK cells, dendritic cells and T cells in viremic controllers and chronically-infected non-controllers with similar CD4^+^ T cell counts in the absence of anti-retroviral therapy. In a multivariable analysis, we identified that retained innate subsets together with preferential Gag-specific over Pol-specific CD8^+^ T cell responses as the best predictors of host control over viral replication in HIV-1 infected viremic controllers compared to chronically-infected non-controllers.

## Materials and Methods

### Subject Criteria and Ethics Statement

25 Uninfected control donors, 15 HIV-1 infected viremic controllers, and 16 chronically-infected non-controllers were enrolled from the greater Philadelphia metropolitan area. All participants provided written informed consent of donation with a comprehensive consent form that is safely stored according to IRB guidelines. Both the study and the consent form were approved by the Institutional review boards of the University of Pennsylvania, Presbyterian Hospital, Drexel University, and The Wistar Institute. All HIV-1 infected controller and non-controller subjects were anti-retroviral therapy naïve or had not received therapy for a period of at least one year prior to recruitment. “Viremic controllers” were defined as having low levels of viral replication (between 50 and 2,000 copies viral RNA/mL). “HIV-1 chronic non-controllers” were recruited with viral loads between 2,000 and 100,000 copies viral RNA/mL. Isolated episodes of viremia above these thresholds were allowed as long as the subsequent visits returned below baseline thresholds. All HIV-1 infected subjects were recruited with CD4^+^ T cell counts above 250 cells/microliter at the time of draw in the absence of anti-retroviral therapy to measure innate and adaptive immune function prior to the loss of function associated with late stage disease progression. Clinical and immune parameters for viremic controllers (median CD4^+^ T cell count of 660 cells/microliter and VL of 704 copies viral RNA/mL) and chronically-infected non-controller subjects (median CD4^+^ T cell count of 554 cells/microliter with viral of 21,932 copies viral RNA/mL) are shown in **Table 1**.

**Table 1:** Cohort Characteristics.

Predictor	Viremic Controllers (n = 15)	Non-controllers (n = 16)	P-value
**VL copies/ml**	704 copies/ml (140–1,354)	21,932 c/ml (7,770–43,280)	*0.0001*
**CD4 Count**	660 cells/microliter (519–971)	554 cells/microliter (455–712)	Not significant
**NK Frequency**	4.51% (3.51–12.7)	2.59% (1.69–5.37)	*0.0276*
**PDC Frequency**	0.17% (0.064–0.229)	0.06% (0.028–0.104)	*0.0168*

### Flow Cytometry

All cell surface antibodies and isotype controls were pre-conjugated and used at the recommended dilution of 0.25 micrograms of antibody per million cells in PBSA (Phosphate buffered saline with 0.09% sodium azide). Peripheral blood mononuclear cells (PBMCs) were stained with antibodies to phenotypic and functional markers for 15 minutes at room temperature in the dark and washed twice. Cells were then fixed and permeabilized with the Cytofix/Cytoperm kit (BD Biosciences, San Jose, CA) and intra-cellular staining was carried out for 15 minutes at room temperature in the dark with 0.25 micrograms of anti-IFN-gamma FITC (BD) per million cells. A minimum of one hundred thousand events were collected on a BD LSR-II Flow Cytometer and samples were subsequently analyzed with FlowJo software (Tree Star Incorporated, Ashland OR). Prior to analysis, all samples were gated by forward (height and area) and side scatter to exclude doublets and dead cells.

### Target Cell-induced NK Degranulation and Intracellular Cytokine Staining Assay

0.5×10^6^ PBMC were co-cultured alone (no target control) or with K562 cells at a 5∶1 effector/target ratio in the presence of 10 microliters anti-CD107a monoclonal antibody, 0.133 microliters of Golgi-stop (BD) and 5 micrograms per milliliter of Brefeldin A (BD) in a 200 microliter volume. A 5∶1 effector/target ratio was chosen to ensure saturation so that every NK cell had access to at least one K562 target cell to induce NK degranulation and cytokine production regardless of the NK frequency per subject. Samples in the presence or absence of target cells were collected at 45 minute intervals for 3 hours. PBMC were washed, stained with antibodies to NK cell phenotypic markers and intra-cellular staining for IFN-gamma was carried out as described above. NK cells were gated by CD56^+^/CD3^−^ expression and the percentage of NK cells staining positive for CD107a degranulation or IFN-gamma production following incubation with K562 cells was determined after subtraction of background levels of staining in the absence of target cells (no target control).

### T Cell Peptide Stimulation and Intracellular Cytokine Staining Assay

0.5×10^6^ PBMC were co-cultured with a 1 microgram per milliliter mixture of overlapping 15-mer peptide pools spanning the HIV-1 Consensus Clade B Gag (123 peptides) or Pol (249 peptides) proteins (AIDS Research and Reference Reagent Repository, NIH) in the presence of 2.5 microliters of CD28/CD49d co-stimulation (BD) and 5 micrograms per milliliter of Brefeldin A for 18 hours in a 200 microliter volume. Alternatively, PBMC were stimulated with a 1 microgram per milliliter mixture of a CEF peptide pool comprising 23 peptides consisting of sequences derived from the human Cytomegalovirus, Epstein-Barr and Influenza Viruses (AIDS Research and Reference Reagent Repository, NIH). Unstimulated and SEB (Staphylococcal Enterotoxin B, Sigma Aldrich) stimulated PBMC (at 5 micrograms per milliliter) were used as negative and positive controls, respectively. PBMC were washed, stained with antibodies to T Cell phenotypic markers and intra-cellular staining for IFN-gamma was carried out as described above. CD8^+^ T cells were gated by CD8^+^/CD3^+^ staining and the percentage of cells staining positive for CD107a and/or IFN-gamma was determined after subtraction of background levels of staining in unstimulated control cells.

### Statistical Analysis

All graphic presentations were performed with Prism software (GraphPad Software, La Jolla, CA) and displayed as median with interquartile range. The following primary variables for analysis (n = 10) were defined a priori to investigate innate and adaptive correlates of control: CD38/HLA-DR activation on CD8^+^ T cells, HIV-1 Gag-specific CD8^+^ T cell responses, HIV-1 Pol-specific CD8^+^ T cell responses, frequency of NK cells, NK cell CD16 expression, NK cell spontaneous CD107a degranulation, NK cell target cell-induced CD107a degranulation, NK cell target cell-induced IFN-gamma cytokine production, PDC frequency, CD40 and CD83 activation and maturation on PDC cells (See **File S1** for all assay parameters and results). Statistical analysis of two groups was carried out using a Wilcoxon-Mann-Whitney test for two independent groups or a Wilcoxon signed-rank test for paired data. Comparisons of three or more groups was carried out using a Kruskal-Wallis test with a post-hoc Dunn test. Correlations between two variables were carried out using Spearman Correlation of untransformed data with a 95% confidence interval. No available data was excluded from analysis as any missing data from groups was the outcome of cell yield or assay limitations. In all cases, significant results have two-sided p values of p<0.05, p<0.01, p<0.001 denoted with a single, double or triple asterisk in graphs, respectively.

To explore the potential best predictors of viral load, a multivariable linear regression analysis using stepwise selection procedure was performed with six target variables (pDC frequency, pDC activation, NK frequency, NK CD16 expression, CD8^+^ T cell activation, and Pol-specific CD8^+^ T cell responses). The graph of predicted versus observed viral load was used to evaluate the ability of model prediction. The residual plot and Breusch-Pagan test were used to examine the heteroskeasticity of the model residuals and an appropriate multivariable linear regression model suggests that residuals are homogeneous.

## Results

### Preferential Gag-specific over Pol-specific CD8 T cell responses are correlated with viral control in viremic controllers versus non-controllers in spite of heightened CD8 T cell activation

**Figure 1:**
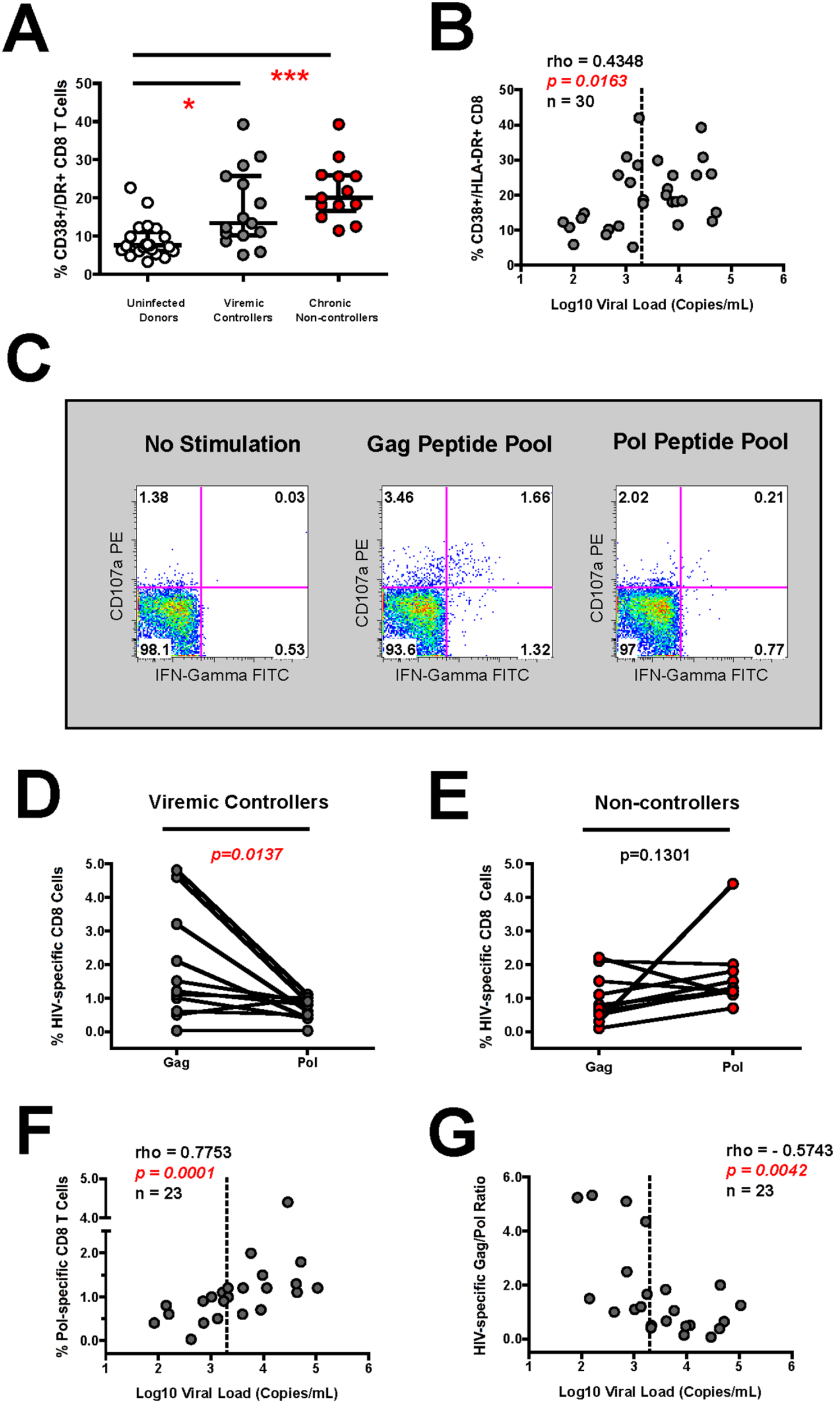
HIV-1 infected viremic controllers possess preferential Gag-specific CD8 T cell responses despite heightened CD8 T Cell activation. (**A**) Composite graph of the percentage of CD38 and HLA-DR activation on CD8^+^ T cells from HIV-1 infected and uninfected subjects. (**B**) Spearman correlation of the percentage of activated CD8^+^ T cells expressing CD38 and HLA-DR (y-axis) with viral load (x-axis) in all HIV-1 infected subjects. (**C**) The HIV-1 specific CD8^+^ T cell response (IFN-gamma production and/or CD107a degranulation) to Gag and Pol peptide pools is shown for a representative HIV-1 infected viremic controller subject. (**D–E**) Composite graph showing the HIV-1 specific CD8^+^ T cell response to Gag and Pol in (**D**) HIV-1 infected viremic controllers and (**E**) chronically-infected non-controllers. (**F–G**) Spearman correlation of the HIV-1 specific CD8^+^ T cell response to a (**F**) Pol peptide pool or (**G**) represented as a Gag/Pol ratio with viral load in all HIV-1 infected subjects. All graphic presentations are displayed as median with interquartile range. Comparisons between two groups were performed using a Wilcoxon matched pairs, non-parametric T test while comparisons between three groups were performed using an unpaired, non-parametric Kruskal-Wallace ANOVA with a Dunn post-test. Correlations between two variables were carried out using a non-parametric Spearman test and dotted line signifies viral load cutoff for viremic controllers (2000 copies/ml or 3.3 Log) In all cases, p-values were two-tailed with a 95% confidence interval and alphas of p<0.05, p<0.01, and p<0.001 are denoted with a single, double or triple asterisk, respectively.

To investigate adaptive immune parameters that correlate with host control over viral replication, we recruited HIV-1 infected viremic controllers (median 704 copies viral RNA/ml) and chronically-infected non-controllers (median 21,932 copies viral RNA/mL) with matched absolute CD4^+^ T cell counts in the absence of anti-retroviral therapy (**Table 1**). Both viremic controllers *(p<0.05)* and chronically-infected non-controllers *(p<0.001)* displayed significantly increased CD8^+^ T cell activation when compared to control uninfected donors (**Figure 1A**). As expected, CD8^+^ T cell activation was also positively correlated with viral load (rho = 0.4348, *p = 0.0163*, n = 30) (**Figure 1B**). We next tested the HIV-specific CD8^+^ T cell response by measuring the capacity of CD8^+^/CD3^+^ gated T cells from fresh PBMC to produce interferon-gamma and/or degranulate in response to overlapping peptide pools comprising the Gag and Pol proteins of HIV-1 (see **Figure 1C** for data from a representative viremic controller). Overall, HIV-1 infected viremic controllers displayed significantly (*p = 0.0137*) increased CD8^+^ T cell preference for Gag-specific responses compared to Pol-specific responses (**Figure 1D**). In contrast, chronically-infected non-controllers displayed reduced Gag-specific responses coupled with increased Pol-specific CD8^+^ T cell responses (**Figure 1E**). Overall, Pol-specific CD8^+^ T cell responses were strongly positively correlated (rho = 0.7753, *p = 0.0001*, n = 23) with viral load (**Figure 1F**), while the Gag-Pol ratio was inversely correlated (rho = −0.5743, *p = 0.0042*, n = 23) with viral load (**Figure 1G**). Of note, HIV-1 infected viremic controllers and chronic non-controllers exhibited similar CD8^+^ T cell antigenic responses to CMV, EBV and Influenza (CEF) as well as CD8^+^ T cell superantigen responses to Staphylococcal Enterotoxin B (SEB) indicating that the overall CD8 T-cell function was retained among subjects in spite of different viral loads (data not shown). Taken together, this data confirms that the preferential targeting of Gag-specific over Pol-specific CD8^+^ T cell responses correlates with virological control in HIV-1 subjects with CD4 counts above 250 cells/microliter in the absence of anti-retroviral therapy.

### Innate parameters distinguish HIV-1 control via retention of NK cell and pDC frequency, NK CD16 expression, and target-cell induced IFN-gamma production but not NK spontaneous degranulation nor target-cell induced degranulation

**Figure 2:**
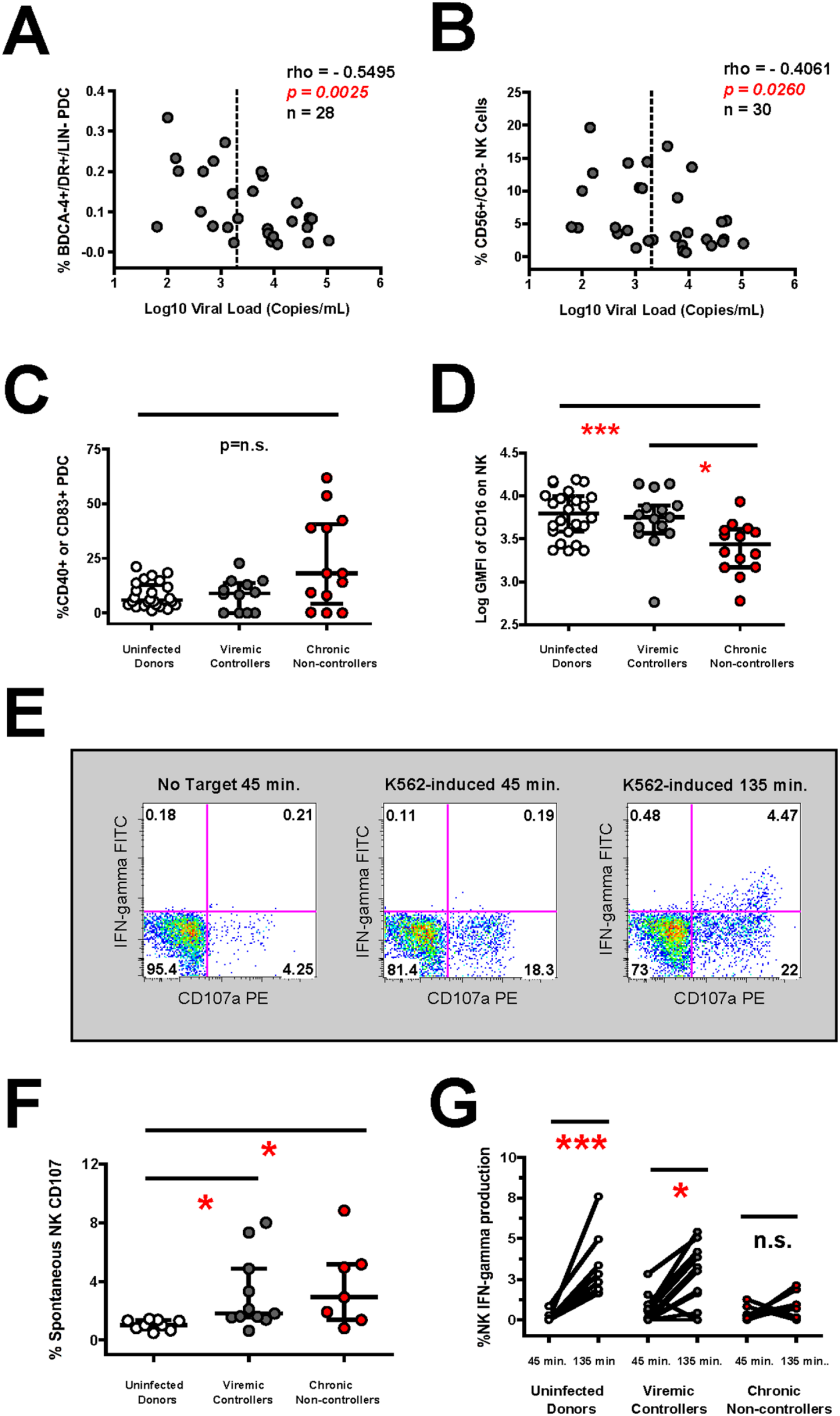
HIV-1 infected viremic controllers possess retained innate immune parameters despite heightened NK spontaneous degranulation. (**A–B**) Spearman correlation of the frequency of (**A**) PDC cells (BDCA-4^+^/HLA-DR^+^/LIN^−^) and (**B**) NK cells (CD56^+^/CD3^−^) (y-axis) with the log viral load (x-axis) in all HIV-1 infected subjects. (**C–D**) Composite graph of (**C**) PDC activation (CD83^+^ and/or CD40^+^ upregulation) and (**D**) NK cell CD16 expression (log geometric mean fluorescence intensity) in HIV-1 infected and uninfected subjects. (**E**) Constitutive and target cell-induced NK cell CD107a degranulation (in presence or absence of K562 cells) is shown for a representative HIV-1 infected viremic controller subject at multiple time points post-incubation. (**F**) Composite graph of the spontaneous NK cell CD107a degranulation in HIV-1 infected and uninfected subjects after culturing *in vitro* for 45 minutes in the absence of target cells. (**G**) Target cell-induced NK cell IFN-gamma production is shown for HIV-1 infected and uninfected subjects at multiple time points post-incubation with K562 cells. Statistical analysis carried out as described in Figure 1.

Having characterized the contribution of the CD8^+^ T cell response in relation to viral control, we next investigated the phenotype and activation status of NK cells and dendritic cells from the innate immune compartment. As shown in **Table 1**, we observed that HIV-1 infected viremic controllers exhibited significantly higher plasmacytoid dendritic cell (pDC) *(p = 0.0168)* and NK cell *(p = 0.0276)* frequencies when compared to chronically-infected non-controllers. Overall, both pDC (rho = −0.5495, *p = 0.0025*, n = 28) and NK cell frequency (rho = −0.4061, *p = 0.0260*, n = 30) was inversely correlated with viral load (**Figure 2A and B**). Next we measured pDC phenotype and observed an increased trend of pDC activation/maturation (as evidenced by CD40 and CD83 upregulation [43], [44]) among non-controllers (**Figure 2C**). However this trend was not statistically significant compared to viremic controllers or uninfected donors. Among NK cells, the mean fluorescence intensity of the FcGamma receptor, CD16, was significantly reduced on chronically-infected non-controllers as compared to uninfected control donors *(p<0.001)* or viremic controllers *(p<0.05)* (**Figure 2D**).

We next used a kinetic assay of NK function to detect differences in constitutive and target cell-induced NK degranulation and cytokine production over time (see **Figure 2E** for data from a representative viremic controller). We observed that spontaneous NK cell CD107a degranulation (in the absence of target cells) was significantly increased in viremic controllers (*p<0.05*) and chronically-infected non-controllers (*p<0.05*) compared to uninfected donors (**Figure 2F**). No difference in target cell-induced NK degranulation was detected between groups (data not shown), although differences were observed in the kinetics and magnitude of target cell-induced NK cell cytokine production. As shown in **Figure 2G**, we observed that the IFN-gamma production was significantly increased over time at the 135 minute time point compared to the 45 minute time point in viremic controllers *(p<0.05)* and uninfected control donors *(p<0.001),* but not in chronically-infected non-controllers. Together, this data indicates that retained innate immune parameters including NK and pDC frequency, NK CD16 expression and target cell induced NK IFN-gamma production are correlates with virological control in HIV-1 subjects with CD4 counts above 250 cells/microliter in the absence of anti-retroviral therapy.

### A combination of innate and adaptive immune parameters best predict viral load in a multivariable regression analysis

**Figure 3:**
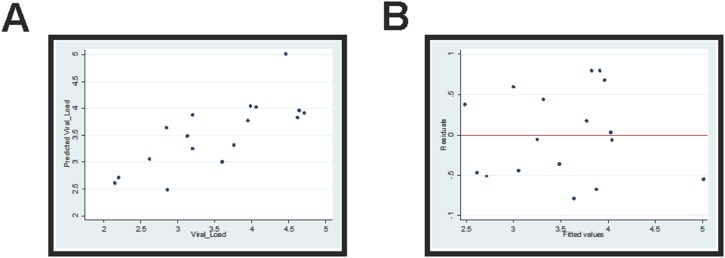
Predicted versus observed viral load with residuals. (**A**) A multivariable linear regression analysis using stepwise selection procedure was performed with six target variables (PDC frequency, PDC activation, NK frequency, NK CD16 expression, CD8^+^ T cell activation, and Pol-specific CD8^+^ T cell responses) to best predict viral load. The graph of predicted versus observed viral load was used to evaluate the confidence in of model prediction. (**B**) A graph of residual values versus fitted values of viral load from the stepwise selection procedure to identify parameters that best predict viral load. The residual plot and Breusch-Pagan test were used to examine the heteroskeasticity of the model residuals and an appropriate multivariable linear regression model suggests that residuals are homogeneous.

Having identified several independent innate and adaptive parameters associated with low viral load in the absence of ART, we generated a multivariable linear regression model to integrate these variables in order to identify the best combination of parameters able to predict viral control. Results of the full multivariable model including all six target variables that were utilized because of their association with viral load (pDC frequency, pDC activation, NK frequency, NK CD16 expression, CD8^+^ T cell activation, and Pol-specific CD8^+^ T cell responses) are shown in **Table 2**. Using stepwise selection procedure, Pol-specific CD8^+^ T cell responses and pDC frequency were the significant predictors (R^2^ = 0.5864, *p = 0.0021, n = 17*) remaining in the final model (**Table 3**). The graph of predicted versus observed viral load has a strong 45 degree pattern in the data (**Figure 3A**) with no obvious pattern observed in the residual plot (**Figure 3B**). However, since the residual plot seemed to slightly expand in the middle, a Breusch-Pagan test was used to further examine the heteroskedasticy. The final model in **Table 3** failed to reject the null hypothesis that residuals are homoskedastic and we thus concluded that residuals are homogeneous (p = 0.4685). Based on our best model, we observed that a combination of innate (pDC frequency) and adaptive (Pol-specific CD8^+^ T cell responses) immune parameters provided the best predictive value for the observed viral load in HIV-1 infected subjects from our cohort.

**Table 2:** Results from Multivariable Linear Regression Model with 6 Predictors of viral load.

Predictor	Estimated coefficient	Lower 95% CI	Upper 95% CI
**CD8 Activation**	0.008	−0.04	0.056
**PDC Frequency**	−3.657	−10.785	3.471
**PDC Activation**	0.006	−0.0218	0.034
**NK Frequency**	−0.015	−0.104	0.074
**NK CD16 Log GMFI**	−0.0001	−0.0002	0.00003
**Pol-specific CD8**	0.309	−0.264	0.882
**Consensus**	3.718	2.643	4.793
**n**	**R-squared**	**Adjusted R^2^**	**F (6, 10)**
**17**	0.6731	0.4769	3.43

**Table 3:** Results of the Final Multivariable Linear Regression Model via Stepwise Selection.

Predictor	Estimated Coefficient	Lower 95% CI	Upper 95% CI	P-value
**PDC Frequency**	−5.84	−9.986	−1.703	*0.009*
**Pol-specific CD8**	0.427	0.108	0.746	*0.012*
**Consensus**	3.630	2.961	4.299	0.000
**n**	**R-squared**	**Adjusted R^2^**	**F (6, 10)**	**P-value F**
**17**	0.5864	0.5273	9.92	*0.0021*

## Discussion

**Figure 4:**
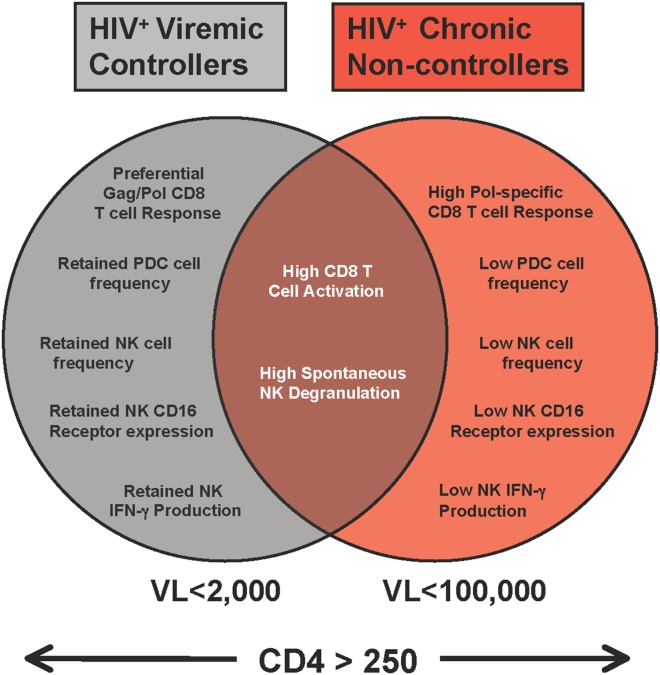
Schematic of the innate and adaptive immune characteristics that distinguish HIV-1 infected viremic controllers from chronically-infected non-controller subjects. Immune characteristics specific to HIV-1 infected viremic controllers (VL<2,000 copies/mL) and chronically-infected non-controller subjects (VL<100,000 copies/mL) with CD4^+^ T cell counts above 250 cells/microliter in the absence of anti-retroviral therapy or common to both are shown in, light gray, red, or dark red, respectively.

Here, we utilized a multivariable analysis to identify which immune parameters on NK cells, dendritic cells and CD8^+^ T cells best correlated with viral control in viremic controllers and chronically infected non-controllers with matched CD4^+^ T cell counts in the absence of ART. Our findings indicate that a higher retention of innate phenotypic and functional parameters (pDC and NK frequency, NK CD16 expression and target cell-induced NK IFN-gamma production) together with preferential CD8^+^ T cell responses targeted towards Gag rather than Pol segregated viremic controllers from chronically-infected non-controllers (see **Figure 4** for our integrated model). In contrast, several variables affected by vial load such as increased CD8^+^ T cell activation and spontaneous NK cell degranulation were inferior in differentiating controllers from non-controllers. In a multivariable analysis, the combination of two variables representing innate (pDC frequency) and adaptive immune parameters (Pol-specific CD8^+^ T cell responses) provided for the best prediction of viral load (**Table 3**), potentially stressing the interplay between innate and adaptive immune compartment in limiting viral replication among viremic controllers.

It is important to stress that our cohort was composed of viremic controller and non-controller subjects with high retained CD4 counts (median CD4^+^ T cell count of 660 cells/microliter and 554 cells/microliter, respectively, **Table 1**) as compared to earlier studies where viral load differences were also associated with end-stage disease [45], [46], [47]. As a result, we did not observe that NK degranulation in response to tumor target cells was inversely affected on a per cell basis by viremia in chronically-infected non-controller subjects from our cohort (data not shown), which is in agreement with previous work measuring NK degranulation during acute infection or chronic viremia [48], [49], [50]. However, we did observe that CD16 expression along with target cell induced NK Interferon-gamma production was decreased in chronically-infected non-controllers compared to viremic controllers from our cohort (**Figure 2D and G**). We also observed that spontaneous degranulation was elevated in both viremic controllers as well as chronically-infected non-controllers (**Figure 2F**). We interpret the increased spontaneous NK CD107a degranulation reflects stimulation *in vivo* that continues *ex vivo* in the absence of target cells. We base this interpretation on our previous work showing that NK cells show high constitutive degranulation over extended periods of time after multiple target cell interactions [51].

Previous studies have shown that there is a higher frequency of viremic controllers possessing protective T cell and NK alleles (such as HLA-B*57 and KIR3DL1*h/*y) than the general population [2], [6], [18], [19], [35], [36]. Our data does not exclude the contribution of genotype toward viral control in subjects from our cohort who control HIV-1 in absence of ART. Rather, we identify joint innate and adaptive immune correlates of HIV-1 control in absence of therapy that inform the type of immune responses that are associated with viral control. We have previously measured the role of protective HLA-B and KIR3DL1*h/*y receptor genotypes in determining the functional state of innate or adaptive immune function in HIV-1 infected controllers [42], and have shown that they are consistent with other studies of HIV-1 infected subjects in general [38], [39], [40].

Our findings here confirm that the presence of a CD8^+^ T cell response directed toward Gag at the expense of other viral proteins like Pol could best distinguish controllers from non-controllers in our study. Recently, both Gag and Pol-specific CD8^+^ T cell responses have been shown to be efficacious in targeting virally infected cells in subjects inheriting protective HLA-B*27 alleles [52]. However, more extensive population based studies have found that Gag-specific, but not Pol-specific, CD8^+^ T cell responses are associated with lower viral loads [26], [27], [28]. In support of those studies, we observed that the Pol-specific CD8^+^ T cell response was associated with increasing viral loads in both a univariable (**Figure 1F**) and multivariable analysis (**Table 3**). We interpret that the observed increase in Pol-specific CD8^+^ T cell responses among non-controller subjects in our study underlie their ineffectiveness in controlling viremia due to the targeting of less sequence constrained epitopes in the Pol protein. In contrast, Gag-specific CD8^+^ T cell responses target conserved epitopes that alter viral interaction with host factors and reduce viral fitness following the emergence of escape mutations [30], [31], [32], [33].

Along with Pol-specific CD8^+^ T cell responses, pDC frequency was identified in the multivariable analysis as the other co-parameter that allowed for the best prediction with viral load (**Table 3**). pDCs have been shown to be redistributed to the lymph nodes and gut mucosa of HIV-1 infected subjects as well as in SIV infected macaques during viremia [53], [54], [55], [56]. However, pDC redistribution to lymphoid organs has also been shown to be associated with increased levels of apoptosis and necrosis among pDCs in SIV infected macaques [57], [58], suggesting that both homing and death of pDC may be responsible for the observed depletion of pDC from the peripheral blood during HIV/SIV disease progression. A loss in pDC frequency and IFN-alpha secretion could have detrimental effects on both NK and CD8^+^ T cell cytolytic responses providing an explanation for its contribution to predicting viral load. Specifically, IFN-alpha production has been shown to be required for NK-mediated lysis of herpes-virus infected target cells [59], [60], [61], [62], [63] and autologous HIV-1 infected primary CD4^+^ T cells [64]. IFN-alpha administration has also been shown to increase perforin expression in both NK cells and CD8^+^ T cells [65], [66]. Importantly, our study raises the hypothesis that retained innate immune parameters such as pDC frequency may also predict immune control over viral replication on anti-retroviral therapy. In support of this hypothesis, pDC levels in HIV-1 infected subjects during anti-retroviral therapy have been associated with viral control upon anti-retroviral therapy interruption [67], [68] and the addition of IFN-alpha while receiving anti-retroviral therapy has been associated with greater viral control [69], [70], [71]. In addition, we have independently observed that a combination of Gag-specific HIV-specific CD3^+^/CD4^−/^perforin^+^/IFN-gamma^+^ cells and the frequency of pDC when measured on anti-retroviral therapy predicted viral set-point after anti-retroviral therapy interruption in 31 subjects (unpublished data).

In conclusion, our data strongly suggest that correlates of viral control among untreated HIV-1 infected subjects may be best evaluated by joint innate and adaptive measures rather than single isolated variables. Future studies will need to test improved models for HIV-1 control by integrating additional variables that may contribute to viral control such as IFN-induced gene expression of host anti-viral proteins (Tetherin, APOBEC, MX2, and TRIM-5 alpha).

## Supporting Information

File S1 (XLS)
